# Supplementary material for: Kinesin motility is driven by subdomain dynamics
Source: eLife. 2017 Nov 7;6:e28948. doi: 10.7554/eLife.28948 (PMC5718755; doi:10.7554/eLife.28948)
Supplement: Supplementary file 1. [file elife-28948-supp1.pdf]

PDB	Nuc.	Conf.	sw-I	$\alpha 4$	L11
Kinesin-1					
1BG2	d	pre	2	-	L
1GOJ	d	post	L	Ext.	L
1MKJ	d	post	2	-	-
2KIN	d	post	2	-	-
2Y5W	d	post	2	-	-
2Y65	d	post	2	-	-
3KIN	d	post	2	-	-
3WRD-A	O	post	2	-	-
3WRD-B	O	post	-	-	-
3X2T	d	post	2	-	-
4HNA	T	post	β	Ext.	1
4LNU	O	pre	-	Ext.	L
5LT0	O	pre	2	-	-
5LT1	O	pre	2	Ext.	-
5LT2	O	pre	β	Ext.	L
5LT3-A–D	O	pre	-	Ext.	L
5LT3-E	O	pre	-	Ext.	1
5LT4	O	pre	β	Ext.	L
Kinesin-2					
2VVG	d	post	-	-	-
3B6U	d	post	2	-	-
3B6V	d	post	-	-	-
Kinesin-3					
1I5S	d	pre	1	Ext.	-
1I6I	T	post	L	-	-
1VFV	T	post	-	-	-
1VFW	T	post	L	-	-
1VFX	T	post	L	-	-
1VFZ	d	pre	1	Ext.	1
2OWM	d	post	β	-	-
2ZFI	d	pre	2	Ext.	1
2ZFJ	d	pre	L	-	-
2ZFK	d	post	L	-	-
2ZFL	d	post	L	-	-
2ZFM	d	post	β	-	-
Kinesin-4					
3ZFC	T	post	β	Ext.	1
3ZFD	T	post	β	Ext.	1
Kinesin-5					
1II6-A	d	pre	1	Ext.	-
1II6-B	d	pre	1	-	1
1Q0B	d	post	1	-	-
1X88	d	post	1	-	-
1YRS	d	post	1	-	-
2FKY	d	post	1	-	-
2FL2	d	post	1	-	-
2FL6	d	post	1	-	-
2FME	d	post	1	-	1
2G1Q	d	post	1	-	-
2GM1	d	post	1	-	1
2IEH	d	post	1	-	-
2PG2	d	post	1	-	-
2Q2Y	d	post	1	-	-

PDB	Nuc.	Conf.	sw-I	$\alpha 4$	L11
Kinesin-5					
2Q2Z	d	post	1	-	-
2UYI	d	post	1	-	-
2UYM	d	post	1	-	-
2WOG-A	d	post	1	-	-
2WOG-B	d	post	1	-	-
2WOG-C	d	pre	1	-	-
2X2R-A	d	post	1	-	-
2X2R-B	d	post	1	-	-
2X2R-C	d	pre	1	-	-
2X7C	d	post	1	-	-
2X7D	d	post	1	-	-
2X7E	d	post	1	-	-
2XAE-A	d	post	1	-	-
2XAE-B	d	post	1	-	-
2XAE-C	d	pre	1	-	-
3CJO	d	post	1	-	-
3HQD	T	post	β	Ext.	1
3K3B	d	pre	1	-	-
3K5E	d	post	1	-	-
3KEN	d	post	1	-	-
3L9H	d	post	1	-	-
3WPN	O	post	-	-	-
3ZCW	d	pre	3	-	-
4A1Z-A	d	pre	1	-	-
4A1Z-B	d	pre	1	-	1
4A28-A	d	pre	1	Ext.	-
4A28-B	d	pre	1	-	1
4A50	d	post	1	-	-
4A51-A	d	post	1	-	-
4A51-B	d	post	1	-	-
4A51-C	d	post	1	-	-
4A51-D	d	post	1	-	-
4A51-E	d	post	1	-	-
4A51-F	d	pre	1	-	-
4A5Y-A	d	pre	1	Ext.	-
4A5Y-B	d	pre	1	-	-
4A5Y-C	d	pre	1	Ext.	-
4AP0-A	d	post	1	-	L
4AP0-B	d	post	1	-	1
4AP0-C	d	post	1	-	-
4AP0-D	d	post	1	-	-
4AS7	d	pre	1	-	2
4B7B	d	pre	1	-	-
4BBG	d	post	1	-	-
4BXN	d	pre	-	-	L
4ZCA-A	d	pre	1	Ext.	-
4ZCA-B	d	pre	1	-	1
4ZHI	d	pre	1	-	-
Kinesin-6					
5X3E	O	pre	L	-	-
Kinesin-7					
1T5C	d	post	1	-	1
2XT3	d	pre	1	-	-
4A14	d	pre	-	-	-

PDB	Nuc.	Conf.	sw-I	$\alpha 4$	L11
Kinesin-8					
3LRE	d	post	L	-	-
Kinesin-9					
3NWN	d	post	1	-	-
Kinesin-10					
3BFN	d	pre	-	-	-
3DC4	d	post	-	-	-
3DCB	T	post	-	-	L
3PXN	d	pre	-	-	-
Kinesin-12					
4BN2	d	post	L	-	L
Kinesin-13					
1RY6	O	pre	1	Ext.	2
1V8J	d	pre	1	Ext.	-
1V8K	T	pre	-	-	-
2GRY	d	pre	-	Ext.	-
2HEH	d	pre	-	-	-
3GBJ-A	d	pre	-	Ext.	-
3GBJ-B	d	pre	-	-	-
3GBJ-C	d	pre	-	-	-
4UBF	d	pre	-	Ext.	-
4Y05	d	pre	-	-	-
Kinesin-14					
1CZ7	d	pre	1	-	-
1F9T	d	pre	2	Ext.	-
1F9U	d	pre	2	Ext.	-
1F9V	d	pre	1	Ext.	L
1F9W	d	pre	-	-	-
1N6M	d	pre	-	-	-
1SDM	d	post	1	-	L
2H58	d	pre	L	Ext.	-
2NCD	d	pre	1	-	-
2REP	d	post	1	-	-
3CNZ	d	post	1	-	-
3COB-A	d	post	1	Ext.	L
3COB-B	d	post	1	-	-
3H4S	d	post	1	Ext.	L
3KAR	d	pre	2	Ext.	L
3L1C-A	d	pre	-	-	L
3L1C-B	d	post	-	-	-
3T0Q	d	post	2	Ext.	L
3U06-A	d	pre	2	-	-
3U06-B	d	post	1	-	-
4ETP	d	pre	3	Ext.	L
4FRZ-A	d	post	-	-	-
4FRZ-B	d	post	L	-	-
4GKR	d	pre	2	-	-
4H1G	d	pre	1	Ext.	-
4OZQ-A	d	post	-	Ext.	L
4OZQ-B	d	post	β	Ext.	-
5HLE	d	pre	1	-	-

Supplementary File 1. Conformational variability of kinesin. Only x-ray structures with resolution better than 3.7 Å are considered (117 PDB structures in total). PDB 4HNA and 4LNU (Kin-1) are the only x-ray structures of kinesin-MT complexes. If chains within the same PDB file differ in conformation, they are listed individually, as for 3WRD-A and 3WRD-B. Nuc.: Nucleotide state (d: ADP, T: ATP-analog, O: APO). Conf.: pre/post-stroke conformations defined based on the relative positioning of $\alpha 4$ and $\alpha 6$ (**??**C,D). For sw-I and L11, number 1–3: approximate number of α-helical turns, L: when they form a mostly visible loop without a clear secondary structure, and '-': mostly invisible. For sw-I, 'β': a pseudo-hairpin. For $\alpha 4$, 'Ext.': the N-terminal side is extended, '-': otherwise. Sw-I takes pseudo-β-hairpin or α-helix conformations, or it is disordered. Similar variations are observed for L11 with no clear relation to the nucleotide state.
