# Supplementary material for: Kinesin motility is driven by subdomain dynamics
Source: eLife. 2017 Nov 7;6:e28948. doi: 10.7554/eLife.28948 (PMC5718755; doi:10.7554/eLife.28948)
Supplement: Supplementary file 2. [file elife-28948-supp2.pdf]

(A) *ATP*

HB Broken	domain	t(ns)	occ
K240-Y307	L11-L14	229.44	0.83
K240-E311	L11-$\alpha 6$	634.80	0.81
D147-S149	L8a-L8a	894.24	0.85
S224-N332	$\beta 7$-NL	918.24	0.81
G76-N332	L3a-NL	920.40	0.91
R190-D231	sw-I-$\beta 7$	1042.56	0.99
T92-D231	$\alpha 2a$-$\beta 7$	1340.16	0.97
E96-R190	$\alpha 2a$-sw-I	1584.00	0.93
E136-H191	$\beta 4$-sw-I	1677.12	0.81
T195-N198	sw-I-sw-I	1825.68	0.97
A193-S201	sw-I-sw-I	1826.16	0.93
N216-Q221	$\beta 6$-L10	2016.72	0.96
R203-E250	sw-I-$\alpha 4$	3593.28	0.86

HB Formed	domain	t(ns)	occ
A83-L232	$\beta 3$-$\beta 7$	1332.00	0.87
E136-R190	$\beta 4$-sw-I	1584.96	0.99
E157-R278	$\beta 5a$-L12	1890.48	0.93
H205-S257	$\beta 6$-$\alpha 4$	2664.00	0.87
I137-N253	$\beta 4$-$\alpha 4$	2758.08	0.81
R190-E250	sw-I-$\alpha 4$	2948.64	0.90
S202-R203	sw-I-sw-I	3900.72	0.84

NP Broken	domain	t(ns)	occ
K213-L335	$\beta 6$-NL	716.16	0.82
N8-K326	CS-NL	910.32	0.97
Y77-V329	L3a-NL	916.08	0.90
L99-I183	L5-$\alpha 3$	1251.36	0.92
V230-L232	$\beta 7$-$\beta 7$	1341.84	0.94
H191-S204	sw-I-sw-I	1676.88	0.85
T195-S201	sw-I-sw-I	1820.40	0.91
V194-E199	sw-I-sw-I	1825.20	0.98
A193-R203	sw-I-sw-I	1833.84	0.98
E215-K222	$\beta 6$-$\beta 7$	2004.96	0.99
E136-S204	$\beta 4$-sw-I	2673.84	0.96
L139-E250	$\beta 4$-$\alpha 4$	2675.76	0.96
Y138-S204	$\beta 4$-sw-I	2688.00	0.99
E250-I254	$\alpha 4$-$\alpha 4$	2883.12	0.80

NP Formed	domain	t(ns)	occ
K91-A233	P-loop-$\beta 7$	1118.64	0.99
I183-K187	$\alpha 3$-$\alpha 3$	1142.88	0.89
K91-D231	P-loop-$\beta 7$	1323.60	0.93
E215-Q218	$\beta 6$-L10	2143.68	0.83
L232-I254	$\beta 7$-$\alpha 4$	2809.92	0.83

(B) *Kin-only*

HB Broken	domain	t(ns)	occ
A5-V329	CS-NL	571.20	0.86
S289-N327	$\alpha 5$-NL	579.36	0.92
G291-N327	L13-NL	579.84	0.91
S289-T296	$\alpha 5$-$\beta 8$	591.36	0.81
D288-N293	$\alpha 5$-L13	619.20	0.86
E136-H191	$\beta 4$-sw-I	1284.72	0.90
R190-D231	sw-I-$\beta 7$	1299.36	0.99
A193-S201	sw-I-sw-I	1302.72	0.95
T195-N198	sw-I-sw-I	1348.80	0.95
D158-R161	$\beta 5a$-L8b	1355.76	0.91
N216-Q221	$\beta 6$-L10	2125.20	0.93
G245-D249	L11-$\alpha 4$	2318.64	0.84
A246-E250	$\alpha 4$-$\alpha 4$	2348.16	0.90
V247-A251	$\alpha 4$-$\alpha 4$	2743.92	0.82

HB Formed	domain	t(ns)	occ
R203-E250	sw-I-$\alpha 4$	544.80	0.96
D288-S289	$\alpha 5$-$\alpha 5$	660.24	0.95
E6-K10	CS-$\beta 1$	930.48	0.93
R190-S206	sw-I-$\beta 6$	1333.68	0.83
R278-A337	L12-NL	1780.08	0.83
M1-D49	CS-$\beta 1c/\beta 2$	2441.28	0.97
D3-R50	CS-$\beta 2$	2443.44	0.99

NP Broken	domain	t(ns)	occ
D288-G292	$\alpha 5$-L13	628.56	0.97
E6-T328	CS-NL	770.88	0.83
K150-L153	L8a-L8a	801.12	0.84
E6-K326	CS-NL	915.60	0.81
T195-S201	sw-I-sw-I	1317.84	0.85
E215-K222	$\beta 6$-$\beta 7$	2191.92	0.86
A243-V247	L11-$\alpha 4$	2307.84	0.86
A246-D249	$\alpha 4$-$\alpha 4$	2341.92	0.89
R50-D64	$\beta 2$-$\alpha 1a$	2448.48	0.94
V247-E250	$\alpha 4$-$\alpha 4$	2754.48	0.84
L248-K252	$\alpha 4$-$\alpha 4$	2898.96	0.81

NP Formed	domain	t(ns)	occ
Y228-L286	$\beta 7$-$\alpha 5$	634.56	0.85
Y228-S289	$\beta 7$-$\alpha 5$	649.68	0.98
N78-S289	L3a-$\alpha 5$	663.36	0.96
R143-K150	$\beta 5$-L8a	864.96	0.89
Y277-T336	L12-NL	2778.48	0.84
Y277-L335	L12-NL	2782.80	0.90
V275-L335	L12-NL	2790.96	0.95
L268-L335	$\alpha 4$-NL	2799.60	0.84

Supplementary File 2. Intra-kinesin hydrogen bonds (HB) and nonpolar (NP) contacts that break or form during each simulation. The corresponding domain names, transition time, and average occupancy (occ) before breakage or after formation of a contact are shown. Only clear transitions were considered where the average occupancy before breakage or after formation is greater than 80%. **(A)** *ATP*, **(B)** *Kin-only*. **Next page: (C)** *ADP+Pi*, **(D)** *ADP$_{pre}$*, **(E)** *APO$_{\alpha}$*, and **(F)** *APO*.
