# Supplementary material for: Kinesin motility is driven by subdomain dynamics
Source: eLife. 2017 Nov 7;6:e28948. doi: 10.7554/eLife.28948 (PMC5718755; doi:10.7554/eLife.28948)
Supplement: Supplementary file 3. [file elife-28948-supp3.pdf]

(C) *ADP+Pi*

HB Broken	domain	t(ns)	occ
R190-D231	sw-I-$\beta 7$	947.28	0.99
H191-R203	sw-I-sw-I	971.52	0.90
T195-N198	sw-I-sw-I	972.00	0.94
N198-H200	sw-I-sw-I	1011.12	0.87
A193-S201	sw-I-sw-I	1042.80	0.98
E36-V51	$\beta 1a/b$-$\beta 2$	1350.96	0.86
E136-H191	$\beta 4$-sw-I	1354.08	0.93
E236-N255	sw-II-$\alpha 4$	1480.56	0.96
N216-Q221	$\beta 6$-L10	2002.56	0.95
Q214-L223	$\beta 6$-$\beta 7$	2632.80	0.87
R203-E236	sw-I-sw-II	5343.84	0.91

HB Formed	domain	t(ns)	occ
E157-R278	$\beta 5a$-L12	169.44	0.99
E136-R190	$\beta 4$-sw-I	1691.52	0.97
V73-G225	$\alpha 1b$-$\beta 7$	2112.96	0.85
E199-R203	sw-I-sw-I	3814.80	0.85
D147-R171	L8a-$\beta 5c$	5136.96	0.89

NP Broken	domain	t(ns)	occ
V194-E199	sw-I-sw-I	977.04	0.97
A193-R203	sw-I-sw-I	1019.76	0.96
T195-S201	sw-I-sw-I	1024.32	0.93
V192-S202	sw-I-sw-I	1345.92	0.82
H191-S204	sw-I-sw-I	1346.40	0.84
E215-K222	$\beta 6$-$\beta 7$	2020.08	0.98
N198-S201	sw-I-sw-I	3717.12	0.87

NP Formed	domain	t(ns)	occ
G76-S224	L3a-$\beta 7$	2109.84	0.83

(D) *ADP_{pre}*

HB Broken	domain	t(ns)	occ
N216-Q221	$\beta 6$-L10	390.00	0.93
H200-S204	sw-I-sw-I	1042.32	0.81
N198-S202	sw-I-sw-I	1054.56	0.80
N196-H200	sw-I-sw-I	1078.32	0.95
M197-S201	sw-I-sw-I	1091.76	0.97
D37-T38	$\beta 1a/b$-$\beta 1b$	2039.52	0.83

HB Formed	domain	t(ns)	occ
Q86-S235	P-loop-sw-II	151.44	0.89
E6-K68	CS-$\alpha 1b$	386.16	0.96
K91-D231	P-loop-$\beta 7$	717.60	0.86
N78-S224	L3a-$\beta 7$	2367.36	0.92

NP Broken	None.

NP Formed	domain	t(ns)	occ
R16-G90	$\beta 1$-P-loop	440.64	0.85
L223-N293	$\beta 7$-L13	2388.00	0.92
N78-L223	L3a-$\beta 7$	2397.60	0.89
V162-K222	$\beta 5b$-$\beta 7$	2420.16	0.91

(E) *APO_{α}*

HB Broken	domain	t(ns)	occ
Q86-S303	P-loop-$\beta 8$	416.64	0.84

HB Formed	domain	t(ns)	occ
Q214-N216	$\beta 6$-$\beta 6$	723.36	0.87

NP Broken	domain	t(ns)	occ
V238-A243	L11-L11	182.88	0.87
E215-K222	$\beta 6$-$\beta 7$	613.92	0.87
L126-N216	$\beta 4$-$\beta 6$	646.56	0.83

NP Formed	domain	t(ns)	occ
V238-K252	L11-$\alpha 4$	191.28	0.94
F128-L223	$\beta 4$-$\beta 7$	702.72	0.81
L126-T219	$\beta 4$-L10	736.08	0.88
V70-Y118	$\alpha 1b$-$\alpha 2b$	762.48	0.93

(F) *APO*

HB Broken	None.

HB Formed	domain	t(ns)	occ
D49-K323	$\beta 1c/\beta 2$-$\alpha 6$	1206.00	0.94
K237-A243	L11-L11	3672.48	0.86

NP Broken	None.

NP Formed	domain	t(ns)	occ
V11-K323	$\beta 1$-$\alpha 6$	1221.84	0.94
Q320-I325	$\alpha 6$-$\alpha 6$	1680.96	0.85
S239-E244	L11-L11	3793.68	0.87

(A) *ATP*

Hbond	Domain	Occ. (time)
S259-αE415	$\alpha 4$	0.95
K237-αE414	L11	0.80
R161-βD427	L8b	0.99
R278-βD427	L12	0.97
E157-βE420	$\beta 5a$	0.89
R278-βE420	L12	0.99 (1886.88)

Nonpolar	Domain	Occ. (time)
K252-αG412	$\alpha 4$	0.97
K256-αV409	$\alpha 4$	0.93
S259-αV409	$\alpha 4$	0.88
S239-αY108	L11	0.85
Y274-βE431	L12	0.91
Y274-βQ434	L12	0.88
H156-βE420	$\beta 5a$	0.85
Y274-βD427	L12	0.84
Y274-βS430	L12	0.83
H156-βM416	$\beta 5a$	0.81
E157-βE420	$\beta 5a$	0.90 (795.60)

(B) *ADP+Pi*

Hbond	Domain	Occ. (time)
S259-αE415	$\alpha 4$	0.97
N263-αR402	$\alpha 4$	0.97
K237-αE414	L11	0.96
K237-αE417	L11	0.91
K313-αE423	$\alpha 6$	0.84
R161-βD427	L8b	1.00
R278-βE420	L12	0.97
E157-βE420	$\beta 5a$	0.96
R278-βD427	L12	0.90
Y274-βE431	L12	0.81 (1497.12)

Nonpolar	Domain	Occ. (time)
S259-αV409	$\alpha 4$	0.99
K256-αV409	$\alpha 4$	0.98
K256-αG410	$\alpha 4$	0.87
S239-αY108	L11	0.84
K252-αG412	$\alpha 4$	0.84
K256-αG410	$\alpha 4$	0.90 (183.60)
H156-βE420	$\beta 5a$	0.99
Y274-βD427	L12	0.94
H156-βM416	$\beta 5a$	0.92
E157-βE420	$\beta 5a$	0.92
Y274-βQ434	L12	0.91
Y274-βE431	L12	0.91
E157-βS423	$\beta 5a$	0.90
R278-βD427	L12	0.86
Y274-βS430	L12	0.84
K159-βT419	$\beta 5a$	0.82

(C) *ADP$_{pre}$*

Hbond	Domain	Occ. (time)
K237-αE417	L11	0.81
R278-βE420	L12	0.98 (728.40)
R284-βE420	$\alpha 5$	0.97 (716.88)
R278-βD427	L12	0.98 {*879.60*}

Nonpolar	Domain	Occ. (time)
K256-αV409	$\alpha 4$	0.95
S259-αV409	$\alpha 4$	0.83
V329-αR422	NL	0.94 (1851.84)
V329-αE423	NL	0.92 (1857.84)
L317-αG416	$\alpha 6$	0.85 (388.32)
Y274-βE431	L12	0.89
Y274-βQ434	L12	0.88
Y274-βS430	L12	0.87
Y274-βD427	L12	0.82

(D) *APO$_{\alpha}$*

Hbond	Domain	Occ. (time)
S259-αE415	$\alpha 4$	0.94
E270-αR402	$\alpha 4$	0.91
K44-αE423	L2	0.90
R321-αE415	$\alpha 6$	0.87
K44-αE420	L2	0.87 (307.44)
S235-αE414	sw-II	0.83 {*159.60*}
R161-βD427	L8b	1.00

Nonpolar	Domain	Occ. (time)
L317-αG416	$\alpha 6$	0.88
Y274-βD427	L12	0.97
Y274-βQ434	L12	0.95
Y274-βE431	L12	0.94
Y274-βS430	L12	0.88

(E) *APO*

Hbond	Domain	Occ. (time)
K237-αE414	L11	0.89
K44-αE423	L2	0.89
D279-βR264	L12	0.99
R161-βD427	L8b	0.99
R278-βN424	L12	0.96
R278-βE196	L12	0.83
R284-βE420	$\alpha 5$	0.90 (2399.76)

Nonpolar	Domain	Occ. (time)
K256-αV409	$\alpha 4$	0.95
S259-αV409	$\alpha 4$	0.94
L248-αY108	$\alpha 4$	0.90
K252-αG412	$\alpha 4$	0.89
L317-αG416	$\alpha 6$	0.85
Y274-βQ434	L12	0.96
Y274-βE431	L12	0.95
Y274-βS430	L12	0.87

Supplementary File 3. Kinesin-MT contacts. For a residue pair in the first column, α and β in front of the second residue denote the tubulin type. Domain: domain name to which the kinesin residue belongs (**??**–Figure Supplement 1). Contacts with α- or β-tubulin are ordered based on occupancy. Contacts whose occupancy after breakage or formation is greater than 80% have the transition time (nanoseconds) in parentheses (formation) or in curly brackets (breakage).
